# Effect of Sand Size on Mechanical Performance of Cement-Based Composite Containing PVA Fibers and Nano-SiO_2_

**DOI:** 10.3390/ma13020325

**Published:** 2020-01-10

**Authors:** Yi-Feng Ling, Peng Zhang, Juan Wang, Yan Shi

**Affiliations:** 1 School of Civil Engineering, Hunan University of Technology, Zhuzhou 412007, China; yling@iastate.edu; 2 National Concrete Pavement Technology Center, Institute for Transportation, Ames, IA 50010, USA; 3 School of Water Conservancy Engineering, Zhengzhou University, Zhengzhou 450001, China; wangjuan@zzu.edu.cn; 4 Changjiang River Scientific Research Institute of Changjiang Water Resources Commission, Wuhan 430010, China; shiyan@mail.crsri.cn

**Keywords:** sand size, PVA fiber, mechanical performance, fracture toughness, nano-SiO_2_

## Abstract

Both finer sand and nanoparticles have a filler effect on mechanical performance of cement-based composite. In this paper, the influence of sand size in mechanical performance of cement-based composites, containing polyvinyl alcohol fiber (PVA) and nano-SiO_2_ (NS), was investigated. The studied mechanical performance, included compressive, flexural, tensile strength, and fracture toughness. A 0.9% volumetric percentage of PVA and a 2% NS mass content were used to make cement-based composites with a 0.38 w/b. Silica sand with four sand size ranges (380–830 μm, 212–380 μm, 120–212 μm and 75–120 μm) was adopted as fine aggregate. The 28-day curing was conducted for all specimens under 20 °C and 95% humidity. It is concluded that the finer sand decreased workability and mechanical strength of PVA-reinforced composites containing NS. However, this reduction was very limited for the sand particles less than 380 µm. The ultimate tensile stain, fracture toughness, and energy were decreased as sand size declined. In addition, the fracture performance of the composites was greatly dependent on fracture energy.

## 1. Introduction

Polyvinyl alcohol (PVA) fiber reinforced cementitious composites have drawn great attention in construction because of their high ductility and excellent fracture performance [1]. PVA fiber significantly increased flexural strength, tensile strength, ultimate tensile strain, fracture energy, and unstable fracture toughness of cementitious composites, while its impact on compressive strength and initiation toughness of cementitious composites was limited. Such toughening effect is associated with bridging effect that reduces stress concentration on cracks [2,3]. Many researchers have reported that PVA fiber can significantly enhance tensile strain capacity, toughness, and the cracking resistance of cementitious composites [4,5]. The volume of PVA fiber varied from 0.5% to 2.0%, and were recommended for use to enhance durability and ductility of cementitious composites [6,7,8]. Sun et al. found that the cementitious composites with 2% PVA fiber content had enhanced strain capacity and toughness [9]. Zhang et al. addressed that 1% PVA fiber content was optimal for reaching maximum mechanical properties of cementitious composites [10]. Even 0.2% PVA fiber content by volume greatly improved the fracture performance of concrete [11]. 

Many researchers have verified that PVA fiber can easily introduce voids into the matrix during mixing and weaken the mechanical performance of composites [12,13]. Moreover, PVA fiber reinforced-cementitious composites need an enhanced bond strength between the cementitious matrix and fibers to ensure sufficient mechanical performance and ductility. Several methods have been used to enhance this interfacial bond, as reported in the literature, which add nanoparticles or finer size sands [14,15]. The nano-particle possess great application prospect in the 21th century. The addition of nano-particle into concrete, not only greatly changed the properties of the fresh concrete, but also improved the mechanical and physical performance, and microstructure of cement-based composites. The nanoparticles are typically used as a cementitious material to strengthen the bond between PVA and matrix. They can promote hydration process and fill pores in cement-based composites to decrease total porosity and enhance mechanical properties [16,17,18]. Gonzalez et al. reported that nano-SiO_2_ (NS) noticeably increased compressive strength and durability of concrete [19]. A 2% NS addition in cementitious composites could increase compressive, tensile and flexural strength by 49%, 38%, and 16% respectively [20,21]. Murthy and Ganesh put forward that the 2% NS addition significantly increased fracture energy of concrete by 30% [22]. A 40% increase in flexural and splitting tensile strength was achieved at 1% NS addition in concrete [23]. Kim et al. observed a decrease in medium capillary pores and an increase in gel pores for the composites containing NS less than 5%, thus enhancing compressive strength [24]. Assaedi et al. concluded that the NS addition accelerated hydration and positively increased the mechanical properties of composites [25]. Thereby, both PVA fibers and NS particles can be applied in cementitious composites to improve the mechanical properties of cementitious composites.

Coarse aggregates are not commonly used in cementitious composites to ensure excellent workability. As PVA fiber-reinforced cementitious composites contain no coarse aggregates and only contain fine aggregates, cementitious materials, and PVA fiber, they need more fine aggregates than traditional concrete [26]. Therefore, sand plays a great role in properties of these composites. In particular, sand size greatly influenced by the flowability of the fresh mixture, mechanical properties, physical properties, and the durability of cementitious composites. Some researchers conducted the study to understand the effect of particle size of sand on the mechanical properties of cement mortar. Kang et al. found that finer sand greatly improved the tensile and flexural strength of mortar because finer sand filled the voids and increased the density of matrix [27]. However, the effectiveness of the reduction of sand size in enhancing mechanical performance of cement-based composites, containing PVA fibers and NS, has still not been fully understood. It needs to be clarified whether there are conflicts or benefits from these two filling effects of nanoparticles and finer sands. The mechanical properties of cementitious composites can be improved, not only by addition of PVA fibers and NS, but also by improving particle size of the sand used. Therefore, in this study, the effect of particle size on sand on mechanical performance of cement-based composite containing PVA fiber and NS was studied. The compressive, flexural, tensile strength, and fracture toughness were tested. Additionally, the flowability of the composites was also evaluated.

## 2. Experimental Program

### 2.1. Materials

The cement meets P.O 42.5 in [28] and has 3.13, and 3266 cm^2^/g in specific gravity and specific surface, respectively. The fly ash used complies with [29]. It has a 2.13 specific gravity and a 2464 cm^2^/g specific surface. The chemical compositions of cementitious materials are shown in Table 1. The basic properties of PVA fiber and NS are presented in Table 2, and Table 3, respectively. The high range water reducer (HRWR) was used to make the cement-based composites properly workable. Its properties are listed in Table 4. The silica sand with four different sizes of 380–830 μm, 212–380 μm, 120–212 μm, and 75–120 μm was employed.

### 2.2. Mix Proportions

In studied cementitious composites, the volumetric percentage of PVA fibers was 0.9%. Four sizes of silica sand (a: 380–830 μm, b: 212–380 μm, c: 120–212 μm and d: 75–120 μm) were used to make cement-based composites. All composites were added by NS at 2% mass percent in binder (cement, fly ash and NS). Thus, four mixes at 0.38 w/c with different sand size were designed. The used mix proportion is shown in Table 5. The four mixes are identified as a, b, c, d, referring to 380–830 μm, 212–380 μm, 120–212 μm, and 75–120 μm, respectively. 

### 2.3. Test Procedures

#### 2.3.1. Mixing

The mixing manner followed ASTM C305-14 [30]. The main solids of matrix, except fiber, were premixed for 2 min. It was added with water and HRWR by 50% for both, followed by 1-min stir, same to the rest. The PVA fibers were added and mixed for 2.5 min each time. All specimens were demolded in one day and moved to a room at 20 °C and 95% relative humidity for 28-day curing.

#### 2.3.2. Slump Flowability

PVA fibers and NS greatly affected workability of cementitious composite [31,32,33]. The slump flowability test was conducted to investigate the effect of sand size on workability of cementitious composites as per ASTM C1611 Procedure A [34]. The average of the two test results per mix was reported for discussion.

#### 2.3.3. Compressive Strength

For each mix, three cubes (70.7 mm × 70.7 mm × 70.7 mm) were prepared to test compressive strength according to JGJ/T70-90 [35]. The average strength of the three duplicates was taken to be reported in discussion.

#### 2.3.4. Flexural Strength

**Figure 1:**
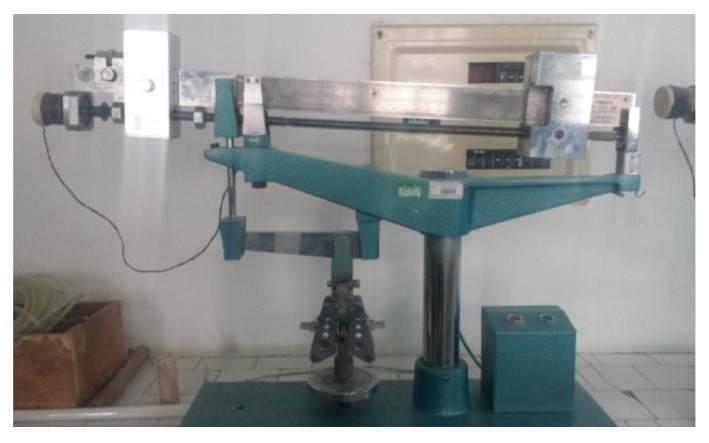
Electric flexure tester.

**Figure 2:**
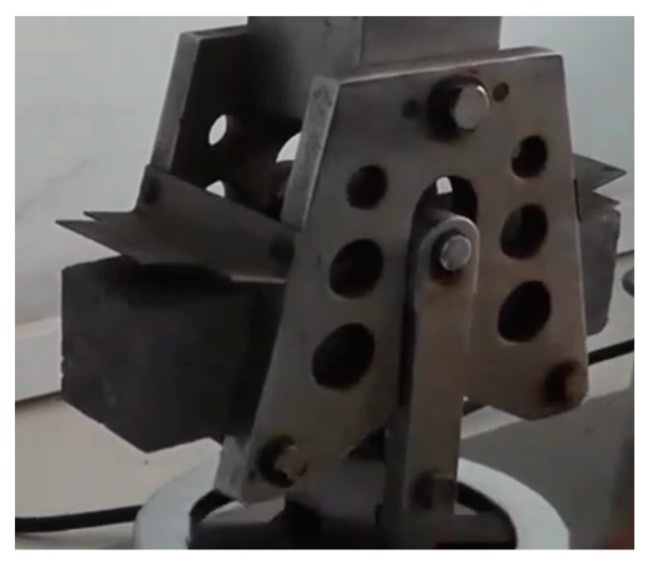
Specimen being fixed in the holder of the electric flexure tester.

Three 40 mm × 40 mm × 160 mm prism specimens were cast for per mix for flexural strength test. The test was conducted in accordance with JTJ E30-2005 [36] using an electric flexure tester, which is shown in Figure 1. Before the test, the tester was adjusted in advance to meet the measuring requirements. Figure 2 presents the specimen being fixed in the holder of the electric flexure tester. After the specimen was fractured, the failure load was recorded from the scale plate. 

The flexural strength (*R*_f_) was calculated by Equation (1),

(1) $$R_{f}=\frac{1.5F_{f}L}{b^{3}}$$

where *F*_f_ is peak load; *L* is support span (100 mm); *b* is prism width (40 mm). The average of three specimens for each mix was reported as flexural strength.

#### 2.3.5. Tensile Strength

**Figure 3:**
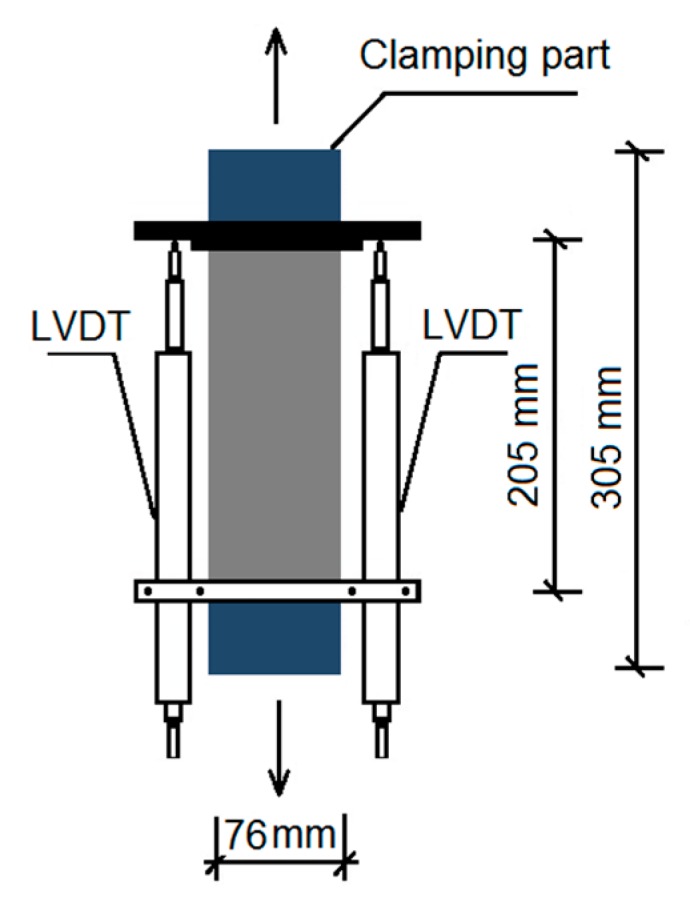
Diagrammatic drawing of the testing device.

The uniaxial tensile test of developed composites was carried out based on JTJ E30-2005 [36]. Three specimens in dimension of 305 mm × 76 mm × 20 mm were cast for each mix. Epoxy resin was applied on two ends of the specimen, which were then wrapped by a carbon cloth to protect the specimen during tensile load. The displacements of specimen under loading were monitored using a linear variable differential transformer (LVDT). The diagrammatic drawing of the testing device is shown in Figure 3. The stress-strain curve was automatically plotted by a computer. The ultimate tensile strain was obtained at half peak load. The reported ultimate strain and strength were the average of three specimens.

#### 2.3.6. Fracture Performance

**Figure 4:**
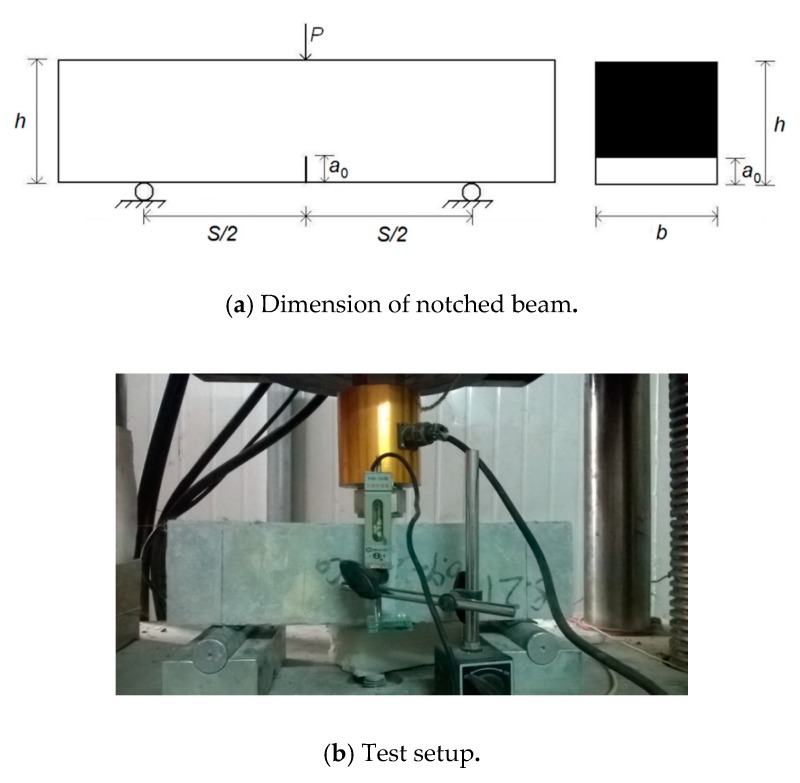
Three-point bending test.

Every mix had three precast notched beams (100 mm×100 mm×400 mm) for three-point bending test to investigate the fracture performance similar to [37]. As presented in Figure 4a, all notched beam specimens had a support span (*S*) of 300 mm, an initial notch length (*a*_0_) of 40 mm, and a notch depth to specimen height ratio (*a*_0_/*h*) of 0.4. The width of notch was 3 mm. Figure 4b shows the setup of test. It can be seen that the deflection at mid span was measured using LVDT. A clip extensometer was embedded in the notch, in order to determine the crack mouth opening displacement (*CMOD*). Loading rate was 0.05 mm/min for the test. The most typical load-mid span deflection and load-*CMOD* (*P*-*CMOD*) curves among three specimens for each mix were selected as result.

The critical *CMOD* was obtained at peak load (*F*_max_). The critical effective crack length (*a*_c_) was determined by Equation (2) based on DL/T5332-2005 [38],

(2) ac=2π(h+h0)arctan(tEVc32.6Fmax−0.1135)12−h0

where *h* is specimen height (0.1 m); *h*_0_ is thickness of clip extensometer (0.001 m); *t* is beam width (0.1 m); *V*_c_ is critical *CMOD* (µm); *E* is elasticity (GPa) expressing in Equation(3),

(3) $$E=\frac{1}{tc_{i}}\left[3.7+32.6\tan^{2}\left(\frac{\pi }{2}\frac{a_{0}+h_{0}}{h+h_{0}}\right)\right]$$

where *c*_i_ is linear slope of *CMOD* versus load curve (µm/kN).

In *P-CMOD*, the initial cracking load (*F*_Q_) is at where the curve changes from linear to non-linear. In accordance with [38], the initial cracking toughness ($K_{IC}^{Q}$) was calculated by Equations (4) and (5),

(4) $$K_{IC}^{Q}=\frac{1.5\times \left(F_{Q}+\frac{mg}{2}\times 10^{-2}\right)\times 10^{-3}\times S\times \sqrt{a_{0}}}{th^{2}}\times f\left(\alpha \right)$$

(5) f(α)=1.99−α(1−α)(2.15−3.93α+2.7α2)(1+2α)(1−α)32, α=a0h

where *m* is specimen weight within mid-span (kg); *g* is gravity acceleration (9.81 m/s^2^).

The unstable cracking toughness ($K_{IC}^{S}$) is thefracture toughness at *F*_max_. As per [38], it can be calculated by Equations (6) and (7):

(6) $$K_{IC}^{S}=\frac{1.5\left(F_{\max}+\frac{mg}{2}\times 10^{-2}\right)\times 10^{-3}\times S\times \sqrt{a_{c}}}{th^{2}}\times f\left(\alpha \right)$$

(7) f(α)=1.99−α(1−α)(2.15−3.93α+2.7α2)(1+2α)(1−α)32,α=ach

According to the double-*K* fracture theory, crack propagation meets:

(a) If $K<K_{IC}^{Q}$, crack will not propagate;
(b) If $K_{IC}^{Q}\leq K\leq K_{IC}^{S}$, crack will stably propagate;
(c) If $K>K_{IC}^{S}$, crack will unstably propagate.

Fracture energy (*G*_F_), a crack resistance index [39], was expressed in Equation (8) based on [40],

(8) $$G_{F\;}=\frac{W_{0}}{A}+\frac{mg\delta_{0}}{A}$$

where *W*_0_ (N∙m) is area of P-CMOD curve; m (kg) is specimen weight between two supports and the load portion; *δ*_0_ (m) is fractured reflection; *A* (m^2^) is net area at fracture that was calculated by Equation (9),

(9) $$A=b\times \left(h-a_{0}\right)$$

where *b* (m) is beam width.

The fracture toughness of cementitious composites was systematically evaluated based on the initial cracking toughness ($K_{IC}^{Q}$), unstable cracking toughness ($K_{IC}^{S}$), and fracture energy (*G*_F_) [41,42].

## 3. Results and Discussion

### 3.1. Slump Flowability

**Figure 5:**
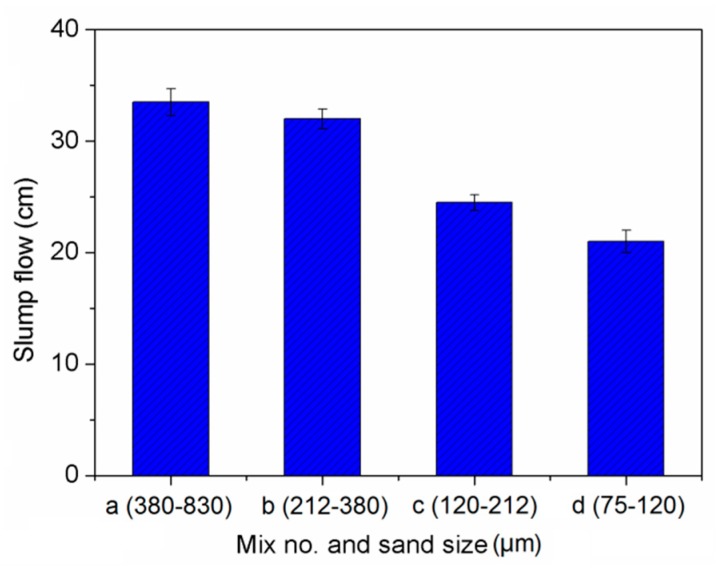
Slump flow of cement-based composites.

The effect of sand size on slump flowability of cement-based composites is shown in Figure 5. It indicates that the slump flowability of Mix b, Mix c, and Mix d were greatly reduced by 5%, 27%, and 37%, respectively with sand size decreasing, compared with Mix a. The whole uniformity of the cementitious composite mixtures were decreased due to the use of PVA fibers and NS particles. With the particle size of sand decreasing, more water was absorbed on the increased specific surface area of sand particles [43]. The surface of PVA fibers absorbed a great deal of free water due to good hydrophilia of PVA fibers. As a result, the smaller particle size of sand reduced the workability of the composites.

### 3.2. Compressive Strength

**Figure 6:**
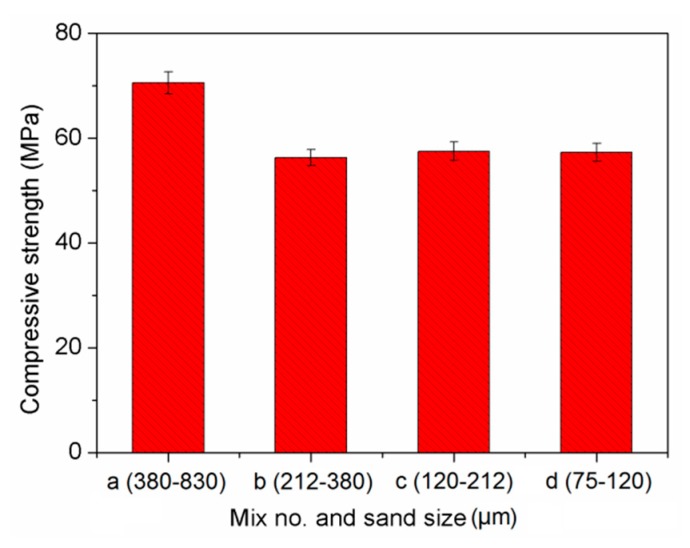
Compressive strength of cement-based composites.

Figure 6 illustrates the effect of sand size on compressive strength of cement-based composites. It can be found that the maximum compressive strength was 70.6 MPa at the largest size of sand (Mix a). The compressive strength was decreased first, and then became stabilized when the sand size was less than 380 µm. Such reduction in strength was due to the finer sands absorbing more water and resulting in insufficient water for hydration. Moreover, the higher amount of fine sand needed more water to secure the workability of composites leading to a reduction in strength [44,45]. Consequently, the water amount used for cement hydration was reduced, which weakened the strength development of cementitious composites.

### 3.3. Flexural Strength

**Figure 7:**
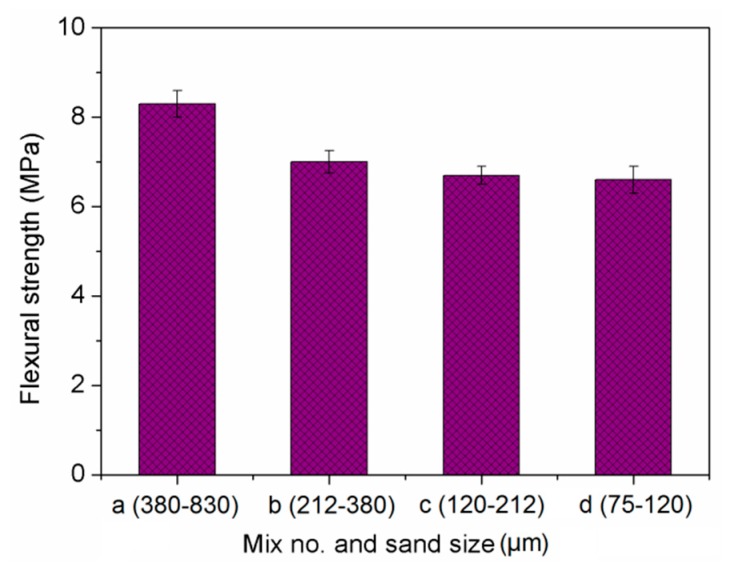
Flexural strength of cement-based composites.

As shown in Figure 7, a smaller sand size also weakened the flexural strength of the composites. From Mix a to Mix d, the flexural strength was reduced by 20% from 8.3 MPa to 6.6 MPa. The trend was similar to the compressive strength. It was expected that the finer sand would increase strength of composite. However, the filler effect of 2% NS significantly reduced that of finer sand on strength [46], therefore, inadequate hydration overwhelmed the filling effect and decreased the strength [47]. Additionally, too much NS flocked together and reduced strength due to large molecular force [48]. Compared to the sand with larger particle size, the sand with smaller particle size had smoother surface, which weakened the bond strength between the sand particles and the binder materials.

### 3.4. Tensile Strength

**Figure 8:**
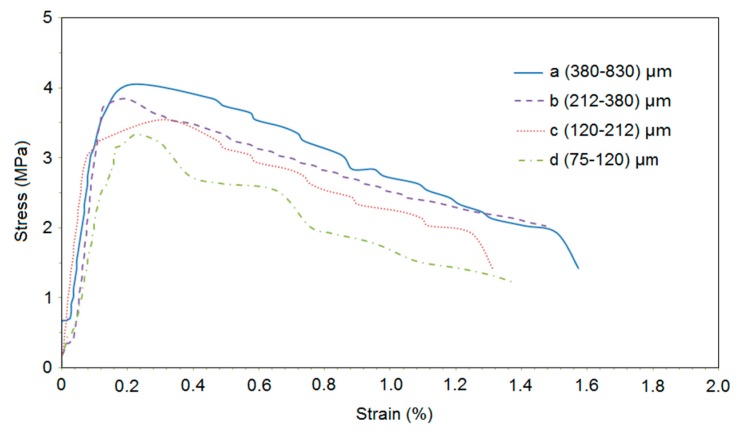
Relationship between stress and strain.

**Figure 9:**
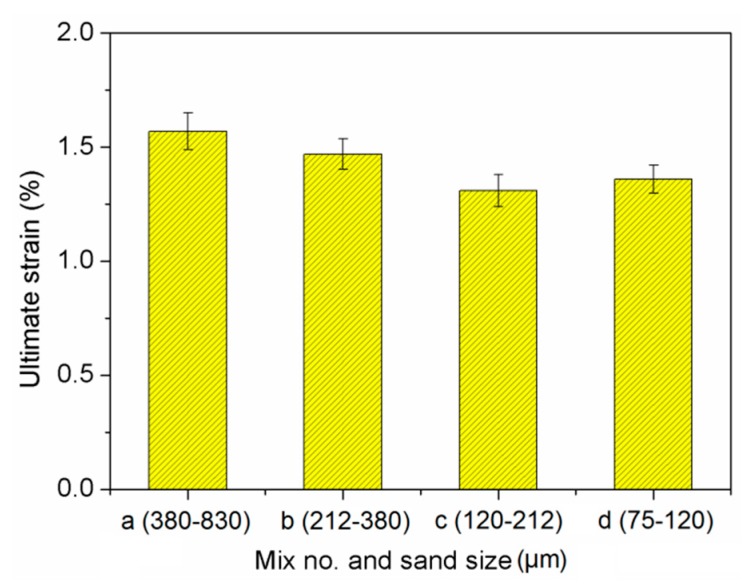
Ultimate strain of cement-based composites.

**Figure 10:**
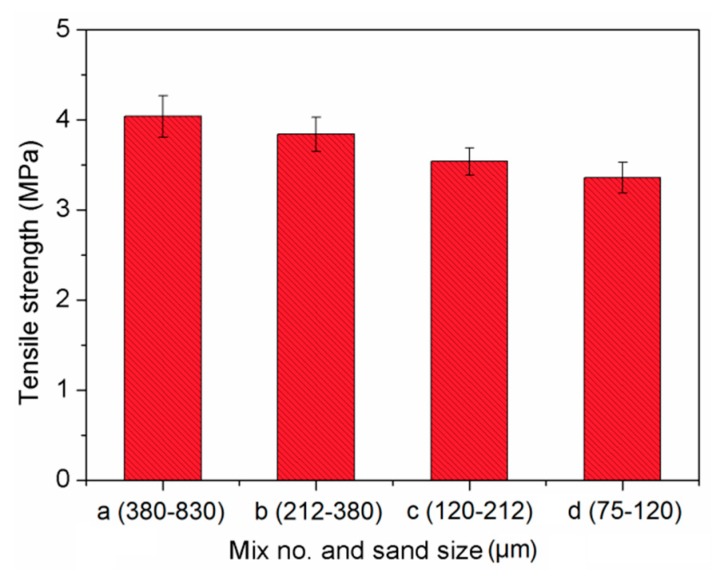
Tensile strength of cement-based composites.

Figure 8 presents the relationship between stress and strain for cement-based composites. Ultimate strength and strain were shown in Figure 9 and Figure 10. The effect of sand size on tensile strength of the composites was consistent with that on compressive and flexural strength. The mechanisms behind this observation are the same as those discussed for compressive strength. It can be seen that the ultimate strain was slightly reduced as sand size decreased. This might be related to a weak bond between sand particles and binder as finer sand particles had a smoother surface than bigger particles [49].

### 3.5. Fracture Performance

**Figure 11:**
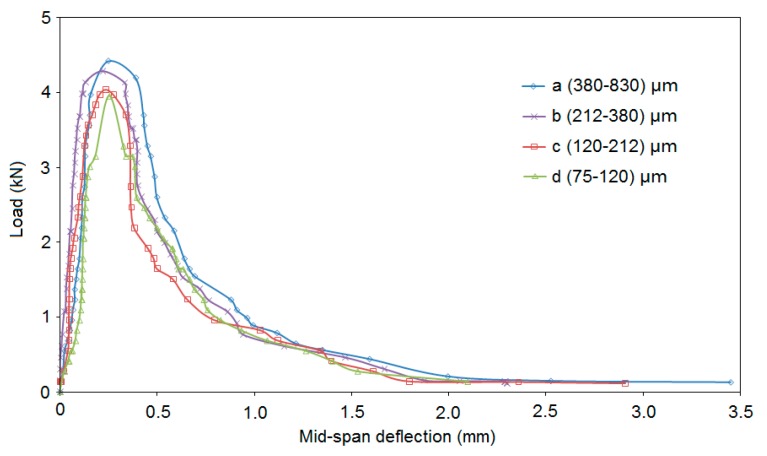
Relationship between load and mid-span deflection.

Figure 11 illustrates load versus mid-span deflection for composites. It shows that the peak load and toughness (area under curve) slightly reduced where sand size decreased, in agreement with those for tensile strength. 

**Figure 12:**
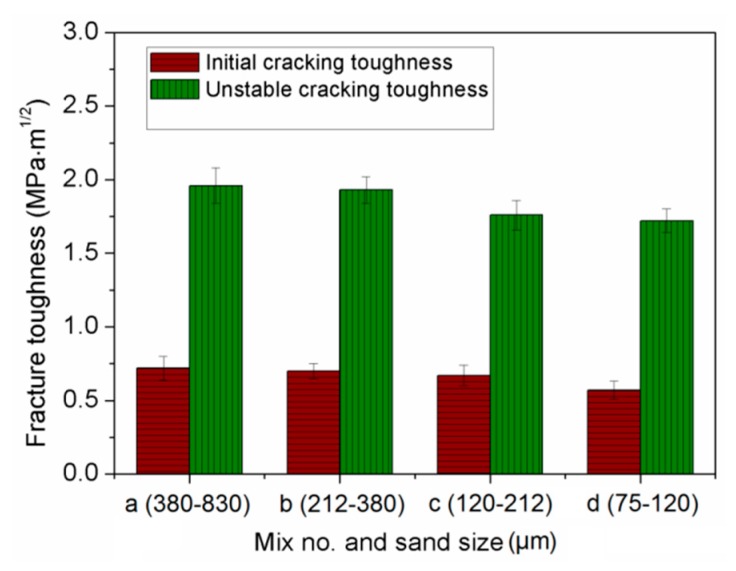
Fracture toughness of cement-based composites.

**Figure 13:**
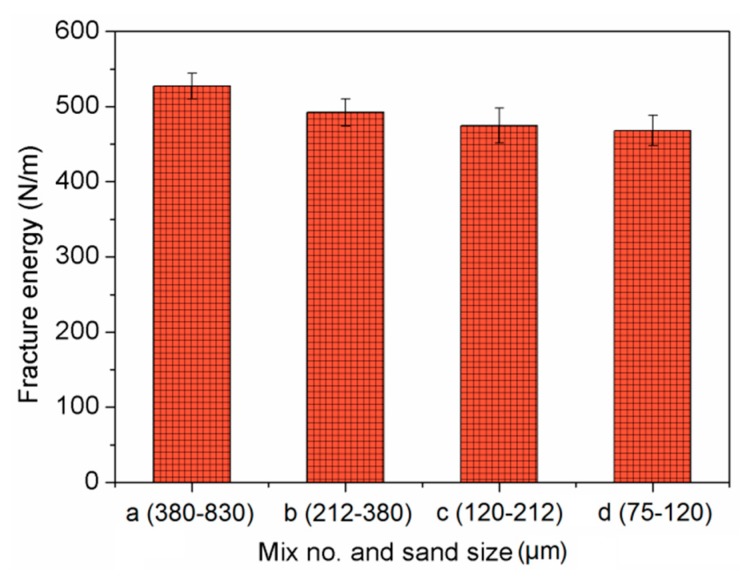
Fracture energy of cement-based composites.

**Figure 14:**
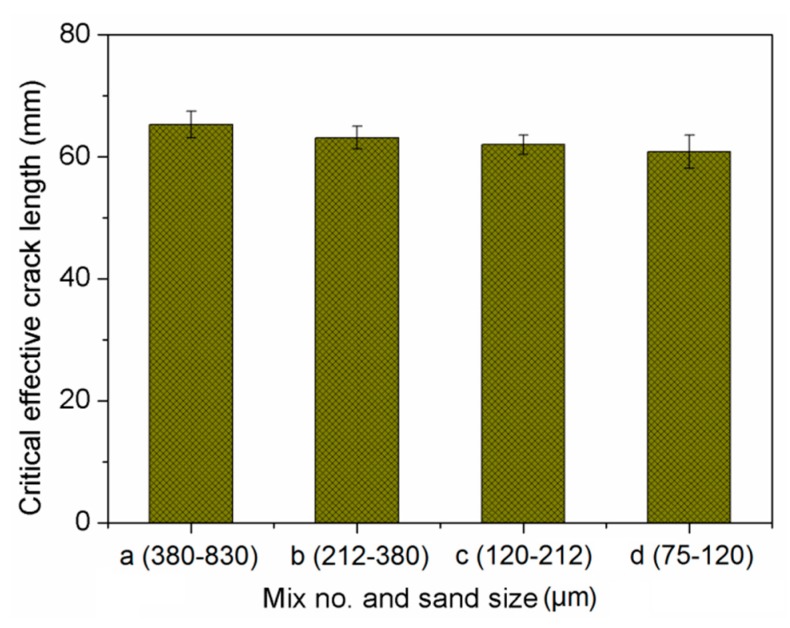
Critical effective crack length of cement-based composites.

**Figure 15:**
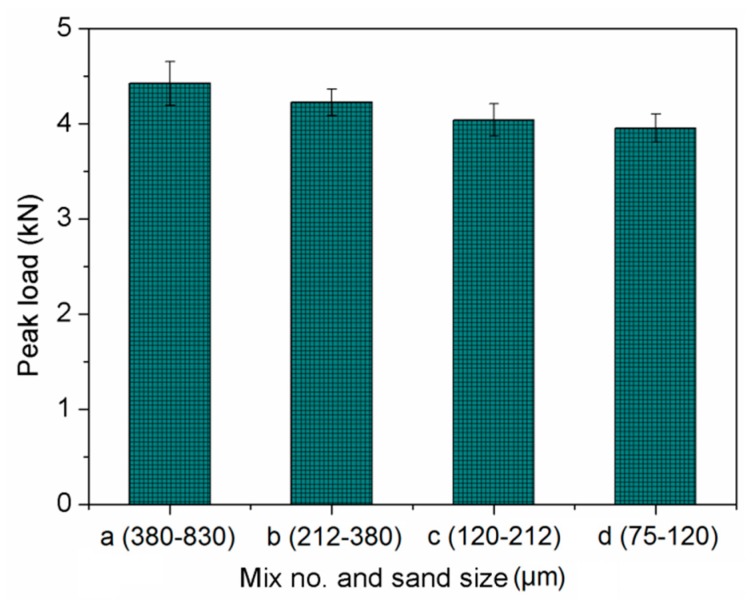
Peak load of specimen of cement-based composites.

The effect of sand size on fracture toughness of composites is shown in Figure 12. It implies that both the initial cracking toughness and unstable cracking toughness declined with decreasing sand size. This phenomenon mirrors the effects on ultimate tensile strain. One explanation refers to fracture energy, as shown in Figure 13. The mix with higher fracture energy has larger fracture toughness. This is associated with the smaller sand particles having lower bond strength between sand and binder [49]. Similar findings were reported in [50]. Based on the obtained mechanical properties, the finer sand in PVA cementitious composites containing NS, not only weakened the strength, but also reduced the ductility. As shown in Figure 14 and Figure 15, the trends of *a_c_*, *F_max_*, and double-*K* fracture toughness were in a good agreement with fracture energy with decreasing sand size. The comparable trends were obtained by Guan et al. [51]. The crack propagation can be continued only on the condition that the crack bypasses the sand particle. The sands with larger particle size have more significant blocking effect on propagation path of the crack, which makes the propagation path more tortuous, and the crack propagation consumes more energy. 

## 4. Conclusions

This paper investigated the effect of sand size on mechanical performance of cement-based composites containing PVA fiber and NS. Based on the experimental results, conclusions were made as follows:

(1) Finer sand significantly reduced the workability of the cement-based composites with PVA and NS.

(2) The cement-based composites were made up of finer sands had lower compressive strength, flexural strength and tensile strength, while such reduction effect was marginal when sand size was less than 380 µm. 

(3) With decreasing sand size, the fracture toughness and ductility of cement-based composites were also reduced.

(4) Fracture energy clearly explained the reduction of fracture toughness caused by finer sand size.

## Figures and Tables

**Table 1:** Composition of cementitious materials.

Composition (%)	Cement	Fly Ash
SiO_2_	21.05	52.12
Al_2_O_3_	5.28	17.86
Fe_2_O_3_	2.57	6.57
CaO	63.14	9.12
MgO	3.58	3.26
Na_2_O	0.17	2.38
K_2_O	0.58	2.05
SO_3_	2.39	0.23

**Table 2:** Basic properties of polyvinyl alcohol (PVA) fiber.

Specific Gravity	Elongation (%)	Length (mm)	Diameter (μm)	Tensile Strength (MPa)	Water Absorption (%)	Melting Point (°C)
1.32	15	9	20	1400	<1	220

**Table 3:** Nano-SiO_2_ (NS) properties.

Size (nm)	Content (%)	Specific Surface (m^2^/g)	Density (g/cm^3^)	PH
30	99.5	200	0.055	6

**Table 4:** High range water reducer (HRWR) properties.

Density	Alkali Percent (%)	PH	Chloride Percent (%)	Fluidity (mm)	Effectiveness (%)
1.06	1.2	4.62	0.078	260	22.0

**Table 5:** Mix proportion of the cementitious composite.

Cement (kg/m^3^)	Fly Ash (kg/m^3^)	NS (kg/m^3^)	PVA Fiber (%)	Sand (kg/m^3^)	Water (kg/m^3^)	HRWR (kg/m^3^)
630	350	20	0.9	500	380	3

## References

[1] Ling Y., Zhang P., Wang J., Chen Y. (2019). Effect of PVA fiber on mechanical properties of cementitious composite with and without nano-SiO_2_. Constr. Build. Mater..

[2] Irshidat M.R., Al-Shannaq A. (2018). Using textile reinforced mortar modified with carbon nano tubes to improve flexural performance of RC beams. Compos. Struct..

[3] Ling Y., Wang K., Li W., Shi G., Lu P. (2019). Effect of slag on the mechanical properties and bond strength of fly ash based engineered geopolymer composites. Compos. Part B.

[4] Yu K., Wang Y., Yu J., Xu S. (2017). A strain-hardening cementitious composites with the tensile capacity up to 8%. Constr. Build. Mater..

[5] Haskett M., Mohamed Sadakkathulla M., Oehlers D., Guest G., Pritchard T., Sedav V., Stapleton B. Adelaide Research and Scholarship: Deflection of GFRP and PVA fibre reinforced concrete beams. Proceedings of the 6th International Conference on FRP Composites in Civil Engineering (CICE2012).

[6] Atahan H., Pekmezci B., Tuncel E. (2013). Behavior of PVA fiber-reinforced cementitious composites under static and impact flexural effects. J. Mater. Civ. Eng..

[7] Zhang Y., Sun W., Li Z., Zhou X., Eddie, Chau C. (2008). Impact properties of geopolymer based extrudates incorporated with fly ash and PVA short fiber. Constr. Build. Mater..

[8] Viswanath P., Thachil E. (2008). Properties of polyvinyl alcohol cement pastes. Mater. Struct..

[9] Sun M., Chen Y., Zhu J., Sun T., Shui Z., Ling G., Zhong H., Zheng Y. (2019). Effect of modified polyvinyl alcohol fibers on the mechanical behavior of engineered cementitious composites. Materials.

[10] Zhang W., Yin C., Ma F., Huang Z. (2018). Mechanical properties and carbonation durability of engineered cementitious composites reinforced by polypropylene and hydrophilic polyvinyl alcohol fibers. Materials.

[11] Cao R., Yang H., Lu G. (2019). Effects of high temperature on the burst process of carbon fiber/PVA fiber high-strength concretes. Materials.

[12] Ekaputri J., Limantono H., Triwulan T., Susanto T., Abdullah M. (2016). Effect of PVA fiber in increasing mechanical strength on paste containing glass powder. Key Eng. Mater..

[13] Topic J., Proseka Z., Indrova K., Plachy T., Nezerka V., Kopecky L., Tesarek P. (2015). Effect of PVA modification on the properties of cement composites. Acta Polytechnica.

[14] Bentur A., Alexander M.G. (2000). A review on the work of the RILEM TC159-ETC: engineering of the interfacial transition zone in cementitious composites. Mater. Struct..

[15] Gurrero P., Naaman A.E. (2000). Effect of mortar fineness and adhesive agents on pullout response of steel fibers. ACI Mater. J..

[16] Bastos G., Patino-Barbeito F., Patino-Cambeiro F., Armesto J. (2016). Nano-inclusions applied in cement-matrix composites: a review. Materials.

[17] NajiGivi A., Abdul Rashid S., Aziz F.N.A., Salleh M.A.M. (2010). Experimental investigation of the size effects of SiO_2_ nano-particles on the mechanical properties of binary blended concrete. Compos. Part B.

[18] Zhao H., Wu X., Huang Y., Zhang P., Tian Q., Liu J. (2019). Investigation of moisture transport in cement-based materials using low-field nuclear magnetic resonance imaging. Mag. Concr. Res..

[19] Gonzalez M., Tighe S., Hui K., Rahman S., Oliveira Lima A. (2016). Evaluation of freeze/thaw and scaling response of nano concrete for Portland cement concrete (PCC) pavements. Constr. Build. Mater..

[20] Zhang L., Ma N., Wang Y., Han B., Cui X., Yu X., Ou J. (2016). Study on the reinforcing mechanisms of nano silica to cement-based materials with theoretical calculation and experimental evidence. J. Compos. Mater..

[21] Gesoglu M., Güneyisi E., Asaad D.S., Muhyaddin G.F. (2016). Properties of low binder ultra-high performance cementitious composites: Comparison of nanosilica and microsilica. Constr. Build. Mater..

[22] Murthy A.R., Ganesh P. (2019). Effect of steel fibres and nano silica on fracture properties of medium strength concrete. Adv. Concr. Constr..

[23] Shafiq N., Kumar R., Zahid M., Tufail R. (2019). Effects of modified metakaolin using nano-silica on the mechanical properties and durability of concrete. Materials.

[24] Kim T., Kim J., Jun Y. (2019). Properties of alkali-activated slag paste using new colloidal nano-silica mixing method. Materials.

[25] Assaedi H., Alomayri T., Shaikh F., Low I. (2019). Influence of nano silica particles on durability of flax fabric reinforced geopolymer composites. Materials.

[26] Che J., Wang D., Liu H., Zhang Y. (2019). Mechanical properties of desert sand-based fiber reinforced concrete (DS-FRC). Appl. Sci..

[27] Kang S.H., Ahn T.H., Kim D.J. (2012). Effect of grain size on the mechanical properties and crack formation of HPFRCC containing deformed steel fibers. Cem. Concr. Res..

[28] (2007). Common Portland Cement.

[29] (2017). Fly Ash Used for Cement and Concrete.

[30] (2014). Standard Practice for Mechanical Mixing of Hydraulic Cement Pastes and Mortars of Plastic Consistency.

[31] Kim S.W., Yun H.D. (2016). Flexural behaviour of reinforced concrete beams strengthened with a composite reinforcement layer: BFRP grid and ECC. Constr. Build. Mater..

[32] Felekoglu B., Tosun-Felekoglu K., Ranade R., Zhang Q., Li V.C. (2014). Influence of matrix flowability, fiber mixing procedure, and curing conditions on the mechanical performance of HTPP-ECC. Compos. Part B.

[33] Wu C., Li V.C. (2017). Thermal-mechanical behaviors of CFRP-ECC hybrid under elevated temperatures. Compos. Part B.

[34] (2018). Standard Test Method for Slump Flow of Self-Consolidating Concrete.

[35] (2009). Standard for Test Method of Performance on Building Mortar.

[36] (2005). Test Methods of Cement and Concrete for Highway Engineering.

[37] Guan J., Hu X., Yao X., Wang Q., Li Q., Wu Z. (2017). Fracture of 0.1 and 2 m long mortar beams under three-point-bending. Mater. Des..

[38] (2005). Norm for Fracture Test of Hydraulic Concrete.

[39] Aydin A.C. (2007). Self compactability of high volume hybrid fiber reinforced concrete. Constr. Build. Mater..

[40] Cao P., Feng D., Zhou C., Zuo W. (2014). Study on fracture behavior of polypropylene fiber reinforced concrete with bending beam test and digital speckle method. Comput. Concr..

[41] Golewski G.L. (2018). Green concrete composite incorporating fly ash with high strength and fracture toughness. J. Clean. Prod..

[42] Golewski G.L. (2018). Effect of curing time on the fracture toughness of fly ash concrete composites. Compos. Struct..

[43] Reynolds C.E., Steedman J.C., Threlfall A.J. (2008). Reynolds’s Reinforced Concrete Designer’s Handbook.

[44] Apebo A.J., Shiwua N.S. (2013). Effect of water-cement ratio on the compressive strength of gravel-crushed over burnt bricks concrete. Civ. Eng. Res..

[45] Cho S. (2013). Effect of silt fines on the durability properties of concrete. J. Appl. Sci. Eng..

[46] Atmaca N., Abbas M.L., Atmaca A. (2017). Effects of nanosilica on the gas permeability, durability and mechanical properties of high-strength lightweight concrete. Constr. Build. Mater..

[47] Haruehansapong S., Pulngern T., Chucheepsakul S. (2014). Effect of the particle size of nanosilica on the compressive strength and the optimum replacement content of cement mortar containing nano-SiO_2_. Constr. Build. Mater..

[48] Abbasi S.M., Ahmadi H., Khalaj G. (2016). Microstructure and mechanical properties of a metakaolinite-based geopolymer nanocomposite reinforced with carbon nanotubes. Ceram. Int..

[49] Marques A.S., Amaral P.M., Rosa L.G., Fernandes J.C. (2010). Study of aggregate size effect on fracture toughness of petreous macrocomposites (concrete). Mater. Sci. Forum.

[50] Guan J., Yuan P., Hu X., Qing L., Yao X. (2019). Statistical analysis of concrete fracture using normal distribution pertinent to maximum aggregate size. Theor. Appl. Fract. Mech..

[51] Guan J., Li C., Wang J., Qing L., Song Z., Liu Z. (2019). Determination of fracture parameter and prediction of structural fracture using various concrete specimen types. Theor. Appl. Fract. Mech..

